# Acute Physiological Response Versus Chronic Clinical Benefit: A Methodological Concern in the Meta‐Analysis by Cheng et al.

**DOI:** 10.1002/clc.70328

**Published:** 2026-04-21

**Authors:** Hira Bhurgri, Areesha Shafqat

**Affiliations:** ^1^ Liaquat University of Medical & Health Sciences (LUMHS) Jamshoro Pakistan; ^2^ Liaquat National Hospital and Medical College (LNHMC) Karachi Pakistan

To the Editor,

Cheng et al. report that voluntary slow breathing exercise (VSBE) is associated with significant reductions in systolic blood pressure (SBP) in patients with hypertension, suggesting potential value as a low‐cost adjunctive intervention [1]. However, an important methodological issue may limit the clinical interpretation of their pooled estimate: the meta‐analysis combines trials assessing fundamentally different physiological states—immediate or short‐session responses alongside longer‐term resting blood pressure outcomes.

As detailed in the meta‐analysis by Cheng et al., included studies varied substantially in duration, ranging from a single 5 min session to 6 months of training. Notably, Srinivasan et al. assessed a 30 min single session, and Mitsungnern et al. evaluated a 3 h acute protocol in an emergency‐setting cohort: a population fundamentally distinct from the mild‐to‐moderate hypertensive patients for whom the authors' clinical recommendations are intended. Pooling acute trials alongside longer training programs raises concern that the overall effect estimate reflects a mixture of transient hemodynamic responses and sustained resting adaptations, rather than a unified chronic antihypertensive effect.

This distinction carries physiological relevance. Prior research has demonstrated that while acute slow breathing reduces muscle sympathetic nerve activity in hypertensive patients, this effect is not sustained following long‐term training, and long‐term slow breathing reduces office but not ambulatory blood pressure [2], suggesting that acute and chronic protocols engage meaningfully different regulatory mechanisms.

This concern is further reinforced by the authors' own observation that longer intervention durations did not consistently produce greater benefit. While they attribute this to adherence decline or modality differences, an equally plausible explanation is that acute and chronic trials were pooled despite measuring distinct biological phenomena. The reported *I* ^2^ of 82% for SBP reflects substantial statistical heterogeneity that likely stems from this underlying clinical heterogeneity; consequently, the pooled estimate may obscure the true chronic antihypertensive effect of VSBE in a manner that conventional *I* ^2^ statistics alone may not adequately address.

Given the clinical importance of distinguishing temporary post‐exercise blood pressure reduction from durable resting blood pressure adaptation, a sensitivity analysis excluding acute and emergency‐setting trials, alongside a meta‐regression examining intervention duration as a moderator variable, would meaningfully clarify the long‐term therapeutic potential of VSBE. Until such analyses are available, the pooled estimate warrants cautious interpretation when applied to chronic hypertension management.

## Data Availability

Data sharing is not applicable to this article as no data sets were generated or analyzed during the current study.
